# Genetic Differentiation of the Western Capercaillie Highlights the Importance of South-Eastern Europe for Understanding the Species Phylogeography

**DOI:** 10.1371/journal.pone.0023602

**Published:** 2011-08-29

**Authors:** Marko Bajc, Miran Čas, Dalibor Ballian, Saša Kunovac, Goran Zubić, Marijan Grubešić, Petar Zhelev, Ladislav Paule, Tine Grebenc, Hojka Kraigher

**Affiliations:** 1 Slovenian Forestry Institute, Ljubljana, Slovenia; 2 Faculty of Forestry, Sarajevo, Bosnia and Herzegovina; 3 Municipality Kupres, Republic of Srpska, Bosnia and Herzegovina; 4 Faculty of Forestry, University of Zagreb, Zagreb, Croatia; 5 University of Forestry, Sofia, Bulgaria; 6 Faculty of Forestry, Technical University, Zvolen, Slovakia; University of York, United Kingdom

## Abstract

The Western Capercaillie (*Tetrao urogallus* L.) is a grouse species of open boreal or high altitude forests of Eurasia. It is endangered throughout most mountain range habitat areas in Europe. Two major genetically identifiable lineages of Western Capercaillie have been described to date: the *southern lineage* at the species' southernmost range of distribution in Europe, and the *boreal lineage*. We address the question of genetic differentiation of capercaillie populations from the Rhodope and Rila Mountains in Bulgaria, across the Dinaric Mountains to the Slovenian Alps. The two lineages' contact zone and resulting conservation strategies in this so-far understudied area of distribution have not been previously determined. The results of analysis of mitochondrial DNA control region sequences of 319 samples from the studied populations show that Alpine populations were composed exclusively of *boreal lineage*; Dinaric populations of both, but predominantly (96%) of *boreal lineage*; and Rhodope-Rila populations predominantly (>90%) of *southern lineage* individuals. The Bulgarian mountains were identified as the core area of the *southern lineage*, and the Dinaric Mountains as the western contact zone between both lineages in the Balkans. Bulgarian populations appeared genetically distinct from Alpine and Dinaric populations and exhibited characteristics of a long-term stationary population, suggesting that they should be considered as a glacial relict and probably a distinct subspecies. Although all of the studied populations suffered a decline in the past, the significantly lower level of genetic diversity when compared with the neighbouring Alpine and Bulgarian populations suggests that the isolated Dinaric capercaillie is particularly vulnerable to continuing population decline. The results are discussed in the context of conservation of the species in the Balkans, its principal threats and legal protection status. Potential conservation strategies should consider the existence of the two lineages and their vulnerable Dinaric contact zone and support the specificities of the populations.

## Introduction

The Western Capercaillie (*Tetrao urogallus* L.) is a grouse species of open boreal or high altitude forests of Eurasia and is endangered throughout most mountain range habitat areas in Europe [1], [2]. Considered to be an umbrella species for mature forest ecosystems, the Western Capercaillie serves as an indicator of sufficiently preserved structures of old coniferous or mixed forests of Norway spruce, silver fir and beech, with patches of abundant acidophilic ground vegetation with bilberry [3]–[7]. Western Capercaillie habitats have changed significantly in recent centuries due to human impacts, through land use and forest management practices [8]–[10] and possibly due to recent global climate change resulting in habitat shrinkage and fragmentation, reduced connectivity and population decline [1], [11], [12]. The effects of anthropogenic habitat disturbance and destruction are particularly pronounced in capercaillie populations in Central Europe, at the southern edge of the species habitat range and, to a lesser extent, in the Alps [11], [13]. Similar negative effects of human activities on Western Capercaillie habitat and populations have also been reported for the Slovenian Alps and mountain ranges of the Balkan Peninsula [14]–[18]. In South-Eastern Europe only populations in the Bulgarian Rhodope, Rila and Pirin Mountains are reportedly stable today [19].

Historically, 12 subspecies of Western Capercaillie have been described, based primarily on differences in morphology and behaviour (Table 1), [20]. The results of genetic analysis, however, raise questions about the validity of currently recognised sub-speciation and suggest that it is very likely overinflated [21], [22].

**Table 1:** The list of Western Capercaillie (*Tetrao urogallus*) subspecies and their distribution ranges [20].

Subspecies	Area of distribution
*Tetrao urogallus aquitanus*; Ingram, 1915	Pyrenees (Spain, France, Andorra)
*Tetrao urogallus cantabricus*; Castroviejo, 1967	Cantabrian Mountains (north-western Spain)
*Tetrao urogallus karelicus*; Lönnberg, 1924	Finland and Russian Karelia
*Tetrao urogallus lonnbergi*; Snigirevski, 1957	Kola Peninsula (Finland, Norway and north-western Russia)
*Tetrao urogallus major*; C. L. Brehm, 1831	Central Europe from Germany and the Alps to south-western Baltic states, western Belarus, the western Carpathians and northern Macedonia (Germany, Austria, Italy, Switzerland, Liechtenstein, France, Slovenia, Croatia, Bosnia and Herzegovina, Serbia, Montenegro, Kosovo, Albania, Czech Republic, Slovakia, Poland, Belarus, Lithuania; possibly also Ukraine and Estonia)
*Tetrao urogallus obsoletus*; Snigirevski, 1937	Northern Russia and Siberia
*Tetrao urogallus pleskei*; Stegmann, 1926	Belarus, northern Ukraine, most European Russia
*Tetrao urogallus rudolfi*; Dombrowski, 1912	Carpathian mountains and Rhodopes (Romania, Bulgaria, Greece, Ukraine)
*Tetrao urogallus taczanowskii*; Stejneger, 1885	Central Siberia, south to Altai and north-western Mongolia (Russia, Kazakhstan, China, Mongolia)
*Tetrao urogallus uralensis*; Menzbier, 1887	Southern Urals and south-western Siberia (Russia, Kazakhstan)
*Tetrao urogallus urogallus*; Linnaeus, 1758	Fenno-Scandia (Finland, Sweden, Norway), Scotland (reintroduced)
*Tetrao urogallus volgensis*; Buturlin, 1907	Central and south-eastern Russia

Specific countries presented in this table in parentheses were listed on the basis of geographic description of subspecies distribution ranges as listed by de Juana [20]. The list of countries is not necessarily entirely accurate and should not be taken as a definitive reference.

Extensive phylogenetic and phylogeographic studies of the Western Capercaillie have been made across Eurasia, in search of evidence of genetic diversification and conservation strategies through appropriate management on a local scale [2], [21]–[23]. Two main genetically identifiable lineages have been defined (*sensu* [2]), the *southern lineage* (*T. u. aquitanus*) and the *boreal lineage* (*T. u. urogallus*), with different ecological adaptation and post-glaciation development [2], [23]. It has been hypothesised that the *southern lineage* originally expanded throughout Europe or at least to Romania and Bulgaria, and that the *boreal lineage* expanded in Asia and North-Eastern Europe [2] or evolved from the *southern lineage* during late Pleistocene glacial periods [23]. During the last glacial maximum, 20,000–18,000 YBP, permanent ice cover in Central and South-Eastern Europe was limited to the Alpine glacier extending eastward into north-western Slovenia [24]. It is possible that minor glaciers covered the few highest mountain peaks of the central and south-eastern Dinarides (Bosnia and Herzegovina, Montenegro, Albania) and the Carpathians (Romania, Slovakia). A belt of tundra surrounded the glaciated areas. Forests extended from southern Slovenia along the Balkan Peninsula eastward to Bulgaria and south-eastward to Greece and western Turkey [25]–[27]. A belt of forests also existed in the area of the southern and western Carpathians. Similar conditions probably also existed during the next-to-last (Riss-Saalian) glacial period (200,000–130,000 YBP) in Europe. Following the last glaciation, 12,000–10,000 YBP [27], extensive demographic and range expansion and haplotype diversification have been suggested for the *boreal lineage*, as evidenced by a star-like phylogeny, low nucleotide and high haplotype diversity [2]. In contrast, the *southern lineage* remained localised at its proposed glacial refugium in Iberia (the Cantabrian Mountains and the Pyrenees) and the Balkans (Bulgaria and the southern Carpathians in Romania) [2], [23], which form the southernmost edge of the species' current habitat range. *Southern lineage* populations have been discussed as glacial relicts and as potentially having a different post-glacial diversification from the *boreal lineage*, which occupies the rest of and, at the same time, the majority of the current capercaillie habitat range, including the Alps [2], [22], [23].

Different habitat suitability indexes [28] have been defined for capercaillie populations in the western Balkan Peninsula (Slovenian Dinarides) when compared with the neighbouring Alpine populations [14]. The different habitat characteristics in the Alpine and the Dinaric regions may form the basis for genetic differentiation of the populations in these areas, which would be in line with the two subspecies (*T. urogallus major* and *T. urogallus rudolfi*) [20] and lineages [2] of Western capercaillie described on the Balkan Peninsula. Possible genetic differentiation and a genetic barrier between different *T. urogallus* lineages in Balkans would probably necessitate implementation of different conservation management strategies for different populations in the region.

Recent findings on *southern lineage* individuals in the Balkans [2] suggest that the region, which served as a glacial refugium during the last glacial maximum [27], is probably very important for understanding the phylogeography of the Western capercaillie. No large-scale genetic analyses of the Western capercaillie populations in the Balkans have been published to date. Mitochondrial DNA haplotyping has the potential to reveal the genetic lineage composition of studied populations, to provide insight into their demographic histories and to help elucidate the evolution of the lineages. We have successfully obtained what is, to the best of our knowledge, the first substantial set of mtDNA control region sequence data for genetic differentiation analysis and comparison of most major Western capercaillie populations in the Balkans and south-eastern Alps.

## Results

**Figure 1:**
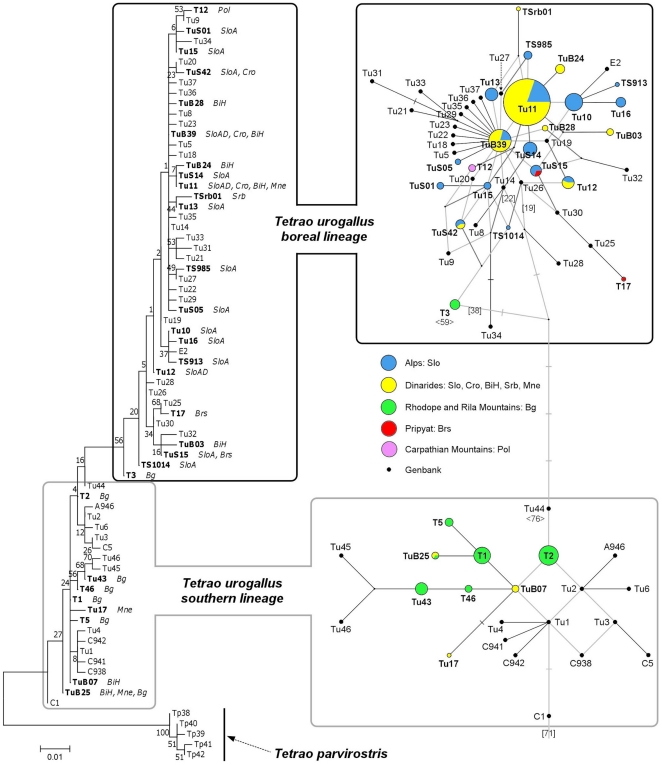
Reconstruction of phylogeny for 68 *Tetrao urogallus* and 5 *Tetrao parvirostris* mtDNA control region haplotypes. A – Maximum likelihood tree according to HKY+G model of nucleotide substitutions with a T_i_/T_v_ ratio of 6.2287 (kappa 11.9429) and gamma distribution parameter alpha of 0.115. Bootstrap support values of 1000-replicate analysis are given for nodes. Western Capercaillie (*Tetrao urogallus*) mtDNA CRI haplotypes discovered in this study in the Balkans and south-eastern Alps are presented in bold. Black-billed Capercaillie (*Tetrao parvirostris*) sequences were used as an outgroup. SloA – Slovenia, Alps; SloD – Slovenia, Dinarides; SloAD – Slovenia, Alps and Dinarides; Cro – Croatia, Dinarides; BiH – Bosnia and Herzegovina, Dinarides; Mne – Montenegro, Dinarides; Srb – Serbia, Dinarides; Bg – Bulgaria, Dinarides; Brs – Belarus, Pripyat; Pol – Poland, Carpathian Mountains. B – Minimum spanning network (using Median-joining algorithm with Maximum parsimony post-analysis). Western Capercaillie (*Tetrao urogallus*) mtDNA CRI haplotypes discovered in this study in the Balkans and south-eastern Alps are presented in bold. Black-billed Capercaillie (*Tetrao parvirostris*) outgroup (linked to haplotype C1) was omitted for clarity. The size of nodes corresponds to haplotype frequencies. Jackknife support, based on a 100-replicate analysis, of the most likely inter-lineage links and C1-to-outgroup link are presented in [ ], values in < > under haplotypes T3 and Tu44 represent jackknife support of their basal-most and terminal-most position within respective lineage haplogroups.

In a total of 319 successfully sequenced Western Capercaillie mtDNA CRI samples collected in Central and South-Eastern Europe (Table 2), we identified 30 unique haplotypes, 21 of which were novel haplotypes, not previously described (Table 3). In the dataset comprising *T. urogallus* haplotypes discovered in this study, there were 28 variable sites (27 substitutions, 1 indel), of which 13 were parsimony informative. For the combined dataset comprising all sampled *T. urogallus* haplotype sequences and *T. urogallus* and closely related Black-billed Capercaillie (*Tetrao parvirostris*) sequences retrieved from Genbank, the best model of nucleotide substitution, chosen according to AICc, was HKY+G. Estimated parameters were: nucleotide equilibrium frequencies of 0.2624, 0.2861, 0.1436 and 0.3079 for A, C, G and T respectively, T_i_/T_v_ ratio of 6.2287 (kappa 11.9429) and gamma distribution parameter alpha of 0.115. ML and MSN methods produced complementary trees (Figure 1) and regrouped the 68 *T. urogallus* haplotypes into two distinct haplogroups: (i) the *boreal lineage* haplogroup was composed of 46 haplotypes, including all Alpine, Polish and Belarus haplotypes, 8 Dinaric haplotypes corresponding to 144 (i.e., 96.00%) samples and 1 Rhodopes-Rila haplotype (T3) corresponding to 6 (i.e., 9.84%) samples; (ii) the *southern lineage* haplogroup was composed of 22 haplotypes including 3 Dinaric haplotypes corresponding to 6 (i.e., 4.00%) samples and 6 Rhodopes-Rila haplotypes corresponding to 55 (i.e., 90.16%) samples.

**Table 2:** Sampling localities, dates and sample sizes of Western Capercaillie (*Tetrao urogallus*) collected in this study, and *T. urogallus* and Black-billed Capercaillie (*Tetrao parvirostris*) mtDNA control region I sequences retrieved from Genbank and used in the phylogenetic analysis.

Pop/seq	Date/Genbank	Species/subspecies	Country	Locality	N	Collector/reference
A	2008–2010	*maj*	Slo	Julian Alps	22	M. Čas, SFS, TNP, SHA
B	2008–2010	*maj*	Slo	Karavanke Mountains	22	M. Čas, SFS, SHA
C	2008–2010	*maj*	Slo	Kamnik-Savinja Alps	46	M. Čas, SFS, SHA
D	2008–2010	*maj*	Slo	Central Eastern Alps	12	M. Čas, SFS, SHA
E	2008–2010	*maj*	Slo	Dinarides	11	M. Čas, SFS, SHA
F	2009–2010	*maj*	Cro	Dinarides	36	M. Grubešić, K. Krapinec
G	2005–2010	*maj*	BiH	Dinarides	92	D. Ballian, S. Kunovac, G. Zubić
H	2009	*maj*	Mne	Dinarides	11	D. Ballian, B. Jokanović
I	1992	*maj*	Srb	Dinarides	1	G. Zubić
J	2009	*rud*	Bg	Rhodopes & Rila Mountains	61	P. Zhelev
K	2009	*ple*	Brs	Prypiat	3	L. Paule
L	2009	*maj*	Pol	Carpathians	3	L. Paule
C1	DQ398967	*can*	Esp	Cantabrians	1	[23]
C5	DQ398971	*can*	Esp	Cantabrians	1	[23]
E2	DQ398961	*maj*	?	Alps	1	[23]
C938	AY750938	*can*	Esp	Cantabrians	1	
C941	AY750941	*can*	Esp	Cantabrians	1	
C942	AY750942	*can*	Esp	Cantabrians	1	
A946	AY750946	*aqu*	?	Pyrenees	1	
Tu1	DQ307392	*can/aqu*	Esp/Fra, And	Cantabrians/Pyrenees	1	[2]
Tu2	DQ307393	*can/aqu*	Esp/Fra, And	Cantabrians/Pyrenees	1	[2]
Tu3	DQ307394	*can*	Esp	Cantabrians	1	[2]
Tu4	DQ307395	*aqu*	Fra	Pyrenees	1	[2]
Tu5	DQ307396	*aqu*	Fra	Pyrenees	1	[2]
Tu6	DQ307397	*aqu*	And	Pyrenees	1	[2]
Tu8	DQ307398	*maj*	Fra	Jura-Alps	1	[2]
Tu9	DQ307399	*maj*	Aus	Alps-Carinthia	1	[2]
Tu10	DQ307400	*maj*	Slo	Alps	1	[2]
Tu11	DQ307401	*maj*	Slo	Alps, Dinarides	1	[2]
Tu12	DQ307402	*maj*	Slo	Alps, Dinarides	1	[2]
Tu13	DQ307403	*obs/maj*	Rus/Slo	Arkhangelsk/Alps	1	[2]
Tu14	DQ307404	*maj*	CzR	Bohemian Mts.	1	[2]
Tu15	DQ307405	*maj/uro*	CzR/Nor	Bohemian Mts./Varaldskogen	1	[2]
Tu16	DQ307406	*maj*	Pol	Carpathians	1	[2]
Tu17	DQ307407	*rud*	Rom	Carpathians	1	[2]
Tu18	DQ307408	*rud*	Rom	Carpathians	1	[2]
Tu19	DQ307409	*rud/ple*, *obs*	Rom/Rus	Carpathians/Tver, Arkhangelsk	1	[2]
Tu20	DQ307410	*maj*	Est	Viljanda	1	[2]
Tu21	DQ307411	*maj*	Est	Viljanda	1	[2]
Tu22	DQ307412	*uro*, *kar*	Fin	Oulu	1	[2]
Tu23	DQ307413	*uro*, *kar*	Fin	Oulu	1	[2]
Tu25	DQ307415	*ple*	Rus	Tver	1	[2]
Tu26	AY580996	*uro*, *kar/ple*, *obs*, *tac*	Fin/Rus	?/Moscow, Tumen, Krasnoyarskii	1	[2], [21]
Tu27	DQ307416	*ple*	Rus	Kaluga, Ivanovo-Poutchege	1	[2]
Tu28	DQ307417	*ple*	Rus	Kaluga	1	[2]
Tu29	DQ307418	*ple*	Rus	Jaroslav'l	1	[2]
Tu30	AY580997	*uro*, *kar/ple*, *obs*	Fin/Rus	?/Jaroslav'l, Arkhangelsk	1	[2], [21]
Tu31	DQ307419	*ple*	Rus	Ivanovo-Poutchege	1	[2]
Tu32	DQ307420	*vol*	Rus	Mordovy Republic	1	[2]
Tu33	DQ307421	*vol*	Rus	Mordovy Republic	1	[2]
Tu34	DQ307422	*obs*	Rus	Arkhangelsk	1	[2]
Tu35	DQ307423	*ura*	Rus	Kirov	1	[2]
Tu36	DQ307424	*obs*	Rus	Tumen	1	[2]
Tu37	DQ307425	*obs*	Rus	Tumen	1	[2]
Tu43	EU030268	*rud*	Bg	Rhodopes	1	[2]
Tu44	EU030269	*rud*	Bg	Pirin Mountains	1	[2]
Tu45	EU030270	*rud*	Bg	Rhodopes	1	[2]
Tu46	EU030271	*rud*	Bg	Rhodopes	1	[2]
Tp38	DQ307426	*T. p. ste*	Mng	Khangayn, Lake Hovsgol	1	[2]
Tp39	DQ307427	*T. p. ste*	Mng	Khangayn	1	[2]
Tp40	AF532463	*T. p. par*	Rus	Magadan	1	[70]
Tp41	AJ297178	*T. p. par*	Rus	Magadan	1	[72]
Tp42	AF532462	*T. p. kam*	Rus	Kamchatka	1	[70]

Pop/Seq – Population/Sequence name; Date/Genbank – Date or period of sampling/Genbank accession number; N – sample size. Only subspecies name codes are listed for *T. urogallus* (*uro – urogallus*, *maj – major*, *can – cantabricus*, *aqu – aquitanus*, *rud – rudolfi*, *ple – pleskei*, *obs – obsoletus*, *kar – karelicus*, *tac – taczanowskii*, *ura – uralensis*, *vol – volgensis*), whereas for *T. parvirostris* first letters of the genus and species name (*T. p.*) precede the subspecies code (*par – parvirostris*, *ste – stegmanni*, *kam – kamschaticus*). Country names are abbreviated (Slo – Slovenia, Cro – Croatia, BiH – Bosnia and Herzegovina, Mne – Montenegro, Srb – Serbia, Bg – Bulgaria, Brs – Belarus, Pol – Poland, Esp – Spain, Fra – France, And – Andorra, Aus – Austria, Rus – Russia, CzR – Czech Republic, Nor – Norway, Rom – Romania, Est – Estonia, Fin – Finland, Mng – Mongolia). SFS – Slovenian Forest Service, TNP – Triglav National Park, SHA – Slovenian Hunting Association.

**Table 3:** Number of individuals per each Western Capercaillie (*Tetrao urogallus*) mtDNA control region I haplotype discovered in sampled populations in the Balkans, south-eastern Alps, Poland and Belarus.

Haplotype	Genbank accession	Population														∑
		A	B	C	D	ABCD	E	F	G	H	I	EFGHI	J	K	L	
Tu10*	HQ852175, DQ307400		1	14	3	**18**										**18**
Tu11*	HQ852176, DQ307401	12	2	9	2	**25**	4	15	76	8		**103**				**128**
Tu12*	HQ852177, DQ307402	1	1		2	**4**	5					**5**				**9**
Tu13*	HQ852178, DQ307403	2	4	4		**10**										**10**
Tu15*	HQ852179, DQ307405	1			2	**3**										**3**
Tu16*	HQ852180, DQ307406	3	1	1	1	**6**										**6**
TuS01	HQ852181	1		1	1	**3**										**3**
TuS05	HQ852182		2			**2**										**2**
TuS14	HQ852183		2	9		**11**										**11**
TuS15	HQ852184		1	4		**5**								2		**7**
TuS42	HQ852185	1	1	1		**3**		2				**2**				**5**
TS913	HQ852186			1		**1**										**1**
TS985	HQ852187		1	2	1	**4**										**4**
TS1014	HQ852188	1				**1**										**1**
TuB03	HQ852189								3			**3**				**3**
TuB24	HQ852190								5			**5**				**5**
TuB28	HQ852191								2			**2**				**2**
TuB39	HQ852192		6			**6**	2	19	2			**23**				**29**
TSrb01	HQ852193										1	**1**				**1**
T3	HQ852194												6			**6**
T12	HQ852195														3	**3**
T17	HQ852196													1		**1**
*TuB07*	HQ852197								3			**3**				**3**
*TuB25*	HQ852198								1	1		**2**	1			**3**
*Tu17* *	HQ852199, DQ852407									1		**1**				**1**
*Tu43* *	HQ852200, EU030268												9			**9**
*T1*	HQ852201												16			**16**
*T2*	HQ852202												22			**22**
*T5*	HQ852203												4			**4**
*T46*	HQ852204												3			**3**
**∑**		**22**	**22**	**46**	**12**	**102**	**11**	**36**	**92**	**10**	**1**	**150**	**61**	**3**	**3**	**319**

A – Slovenia, Julian Alps; B – Slovenia, Karavanke Mountains; C – Slovenia, Kamnik-Savinja Alps; D – Slovenia, Central Eastern Alps; E – Slovenia, Dinarides; F – Croatia, Dinarides; G – Bosnia and Herzegovina, Dinarides; H – Montenegro, Dinarides; I – Serbia, Dinarides; J – Bulgaria, Rhodope and Rila Mountains; K – Belarus, Pripyat; L – Poland, Carpathian Mountains. Columns ABCD and EFGHI represent the cumulative of sampled Alpine and Dinaric populations, respectively. *Boreal lineage* haplotypes are presented in normal font, *southern lineage* haplotypes are presented in italic.

* - haplotypes detected in this study that were identical to already described haplotypes were named as per the original publication with the original Genbank accession number appearing as the second of the two listed.

The *boreal lineage* exhibited a star-like topology of the MSN tree radiating from a central haplotype (TuB39) (Figure 1), which is characteristic of demographic expansion. All haplotypes within the *boreal lineage* haplogroup differed from TuB39 by 1 to 4 mutations. The *southern lineage*, which differed from the *boreal lineage* by 6 mutations (T3-Tu44 link) (Figure 1) consisted only of haplotypes of individuals from two discrete areas at the southern edge of the species' current habitat range (the Pyrenees and the Cantabrian Mountains of Spain, France and Andorra, and the Rhodope-Rila-Pirin Mountains, the Dinarides and the Romanian southern Carpathians in the Balkans) and had a more resolved MSN topology, with longer branches, consistent with long-term stationary populations. No *southern lineage* haplotype exhibited a central position.

The highest haplotype diversity was found for the Alpine group of populations (Table 4). Nucleotide diversities were generally low, with the highest value for Rhodopes-Rila and the lowest for Dinarides. The Dinaric group of populations also exhibited the lowest haplotype diversity, which was about half of the neighbouring Alpine and Rhodopes-Rila populations and the highest observed homozygosity.

**Table 4:** Genetic diversity indices for the mtDNA control region I of Western Capercaillie (*Tetrao urogallus*) populations sampled in the Balkans and south-eastern Alps.

Population	n	*k_OBS_*	*k_EXP_*	S	*Hom*_OBS_	π ± SD	H ± SD	Θ_S_ ± SD	Θ_Π_ ± SD	Θ_Pres_
Alps	102	15	18.20	13	0.1194	0.0044±0.0028	0.8806±0.0169	0.0056±0.0021	0.0044±0.0028	0.0118
Dinarides	150	11	4.62	19	0.4953	0.0037±0.0025	0.5047±0.0459	0.0078±0.0025	0.0037±0.0025	0.0095
Dinarides-A	144	8	4.09	11	0.5372	0.0015±0.0013	0.4628±0.0467	0.0048±0.0018	0.0018±0.0015	0.0082
Rhodopes-Rila	61	7	7.51	12	0.2246	0.0057±0.0035	0.7754±0.0308	0.0057±0.0022	0.0057±0.0035	0.0044

n - Number of samples, *k_OBS_* – observed number of different haplotypes, *k_EXP_* – expected number of different haplotypes, S – number of polymorphic sites, *Hom*_OBS_ – observed homozygosity, π – nucleotide diversities, H – haplotype diversities, Θ_S_ – population parameter theta estimated from the number of segregating sites per nucleotide, Θ_Π_ – population parameter theta estimated from nucleotide diversity per nucleotide and Θ_Pres_, SD – standard deviation. Dinarides-A represents only *boreal lineage* individuals of the combined Dinaric samples for testing the theory of expansion of this lineage in the region in the post-glacial period.

**Figure 2:**
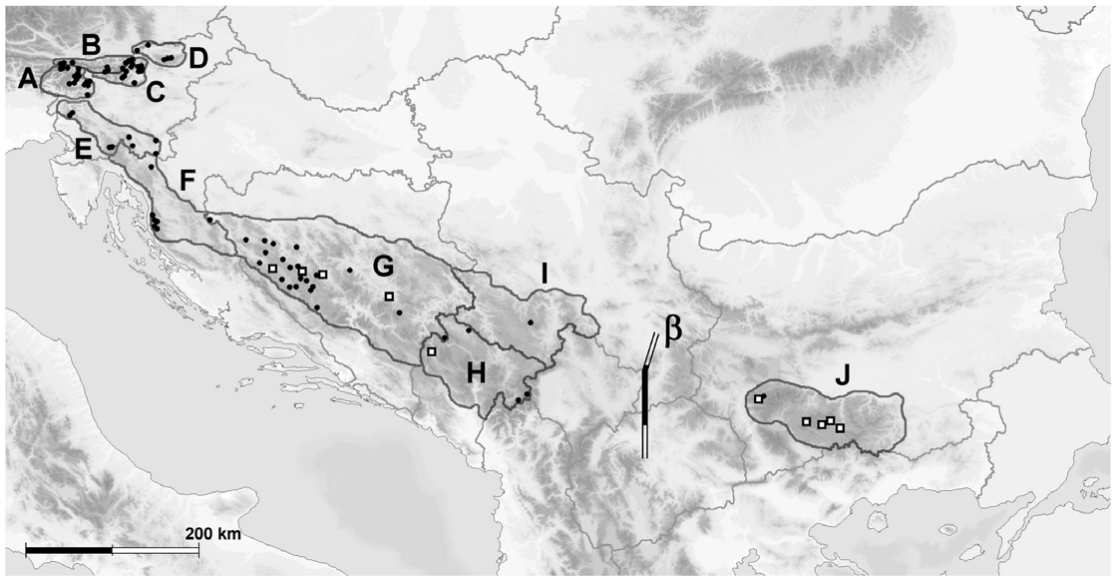
Map of Western Capercaillie (*Tetrao urogallus*) sampling localities in the Balkans and south-eastern Alps. Population codes: A – Slovenia, Julian Alps; B – Slovenia, Karavanke Mountains; C – Slovenia, Kamnik-Savinja Alps; D – Slovenia, Central Eastern Alps; E – Slovenia, Dinarides; F – Croatia, Dinarides; G – Bosnia and Herzegovina, Dinarides; H – Montenegro, Dinarides; I – Serbia, Dinarides; J – Bulgaria, Rhodope and Rila Mountains. Black dots mark the localities where only *boreal lineage* individuals were discovered, white square markers in black outline represent localities where also *southern lineage* individuals were discovered. The line marked β represents the only major genetic barrier according to Monmonier's maximum difference algorithm implemented in Barrier v2.2 software (Results).

According to the pairwise F_ST_ test using the Tamura-Nei model of nucleotide substitutions, the Rhodopes-Rila group appeared distinctly differentiated from both other population groups with statistically significant (p<0.05) pairwise F_ST_ values of 0.7867 for Rhodopes-Rila vs. Alps, 0.7988 for Rhodopes-Rila vs. Dinarides and 0.0548 for Alps vs. Dinarides population pairs. Similarly, the Exact test of population differentiation supported the separation of the three sampled geographic groups with statistical significance (p<0.05). The estimated level of differentiation between Alpine and Dinaric populations was an order of magnitude lower when compared to Alps vs. Rhodopes-Rila or Dinarides vs. Rhodopes-Rila, suggesting a high level of connectivity between Alpine and Dinaric populations in the past. The low level of population differentiation between Alpine and Dinaric populations was also supported by the number of shared haplotypes (Tu11, Tu12, TuS42 and TuB39), representing 37.25% and 88.67% of samples for the Alpine and Dinaric groups, respectively. In contrast we found no shared haplotypes between the Alpine and Rhodopes-Rila groups of populations and a single shared haplotype for the Dinaric and Rhodopes-Rila groups (TuB25), representing only 1.33% and 1.64% of samples, respectively. Population group differentiation estimates were consistent with corrected average number of pairwise differences between population groups (Table 5). Monmonier's maximum difference algorithm, however, revealed only two major groups of populations: one including all Alpine and Dinaric demes and the other including all Rhodopes-Rila samples. The location of the putative genetic barrier between these two groups is presented in Figure 2. According to AMOVA, 79.2% of variance was explained by the variation between groups (Alps/Dinarides vs. Rhodopes-Rila), 16.3% by variation within populations and 4.5% by variation between populations within groups. Results of all of the population differentiation tests and AMOVA were consistent with the proposed geographic distribution of capercaillie subspecies in the region: *T. urogallus major* for the Alps and Dinarides, *T. urogallus rudolfi* for the Rhodopes-Rila mountains [20].

**Table 5:** Average number of pairwise differences assuming Tamura-Nei distances between mtDNA control region I haplotypes of Western capercaillie (*Tetrao urogallus*) populations from the Balkans and south-eastern Alps above diagonal, within populations on the diagonal (in bold) and corrected between population values below diagonal.

	Alps	Dinarides	Rhodopes-Rila
Alps	**1.8002** *	1.7568*	9.4884*
Dinarides	0.0944*	**1.5246** *	8.9435*
Rhodopes-Rila	7.4082*	7.0011*	**2.3602** *

* – statistically significant, p<0,05.

**Figure 3:**
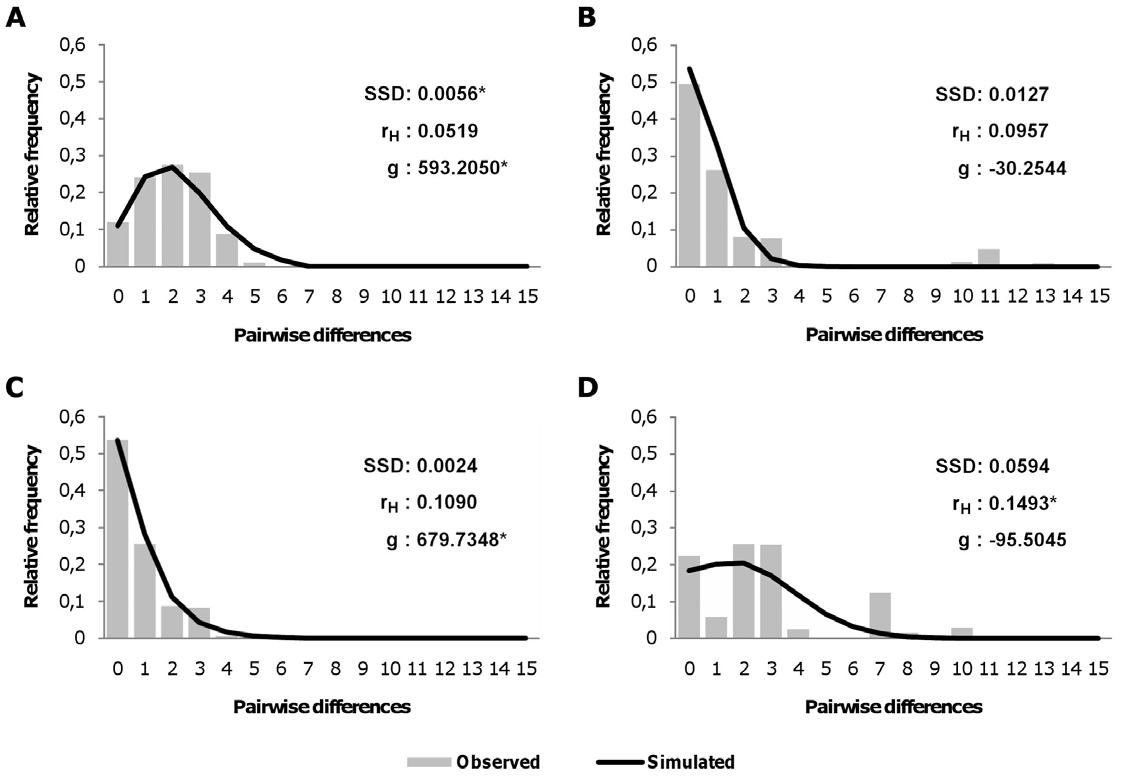
Observed mismatch distributions and their fit to expected model of demographic expansion. Mismatch distributions considering all of the differences, including substitutions and indels, between mtDNA CRI haplotypes of Western Capercaillie (*Tetrao urogallus*) sampled in this study in the Balkans and south-eastern Alps. A – Alps. B – Dinarides. C – only *boreal lineage* individuals of the combined Dinaric samples for testing the theory of expansion of this lineage in the region in the post glacial period. D – Rhodope and Rila Mountains. SSD - Sum of Squared Deviations, r_H_ - Harpending's raggedness index, g - growth force parameter. * – statistically significant, p<0.05.

We examined mismatch distributions of all three geographic sample groups to assess their post-glacial demographic histories. Expanding populations are expected to exhibit an excess of low frequency mutations resulting in unimodal and generally smooth mismatch distribution. Stationary populations, on the other hand, generally exhibit multimodal and rough mismatch distribution due to genetic drift and stochastic lineage loss resulting in fewer more diverged haplotypes of higher frequencies. We assessed the fit of our data to the simulated mismatch distribution under demographic expansion with Harpending's raggedness index and SSD where a nonsignificant test indicates a good fit and support of expansion. Due to the aforementioned excess of low frequency mutations when compared to the expected numbers under the neutrality model, demographic expansion can also be detected through neutrality tests, such as Tajima's D and Fu's Fs, where significant and negative enough values are an indicator of population expansion. Additionally, the growth force parameter g was calculated as an alternative measure of population dynamics. The mismatch distribution for the Alpine population group was unimodal and smooth (Figure 3A) suggesting that it had undergone a demographic expansion. Demographic expansion of the Alpine population was further supported by a low and statistically insignificant Harpending's raggedness index, a low SSD value and high and a statistically significant growth force parameter (Table 6). Tajima's D and Fu's Fs were negative but low, thus the neutrality model could not be rejected.

**Table 6:** Indices used for assessment of demographic status of Western Capercaillie (*Tetrao urogallus*) populations from the Balkans and south-eastern Alps and testing the fit of mismatch distributions to a simulated model of demographic expansion.

	D	Fu's Fs	r_H_	SSD	g
Alps	−0.6008	−4.6531*	0.0519	0.0056*	593.2050#
Dinarides	−1.4552*	−2.0448	0.0957	0.0127	−30.2544
Dinarides-A	−1.5534*	−2.8526	0.1090	0.0024	679.7348#
Rhodopes-Rila	−0.0349	1.9676	0.1493*	0.0594	−95.5045

D - Tajima's D, r_H_ - Harpending's raggedness index, SSD - Sum of Squared Deviations, g - growth force parameter.

* – statistically significant, p<0,05.

# – statistically significant according to exclusion of zero in 95% confidence interval assuming Bonferroni correction.

For the Dinaric population group, the mismatch distribution was bimodal (Figure 3B) as a result of the presence of haplotypes of both lineages: the low-end mode corresponding to within-lineage comparisons and the high-end mode corresponding to between-lineage comparisons. Harpending's index was higher, but insignificant, SSD was higher and significant, suggesting a poor fit to the proposed demographic expansion model. Tajima's D and Fu's Fs were negative (Table 6) but not large enough or statistically significant to reject neutrality. A significant Chakraborty's population amalgamation test [29] (data not shown) indicated that the present Dinaric population had possibly been formed by the amalgamation of two distinct populations. The Result of Chakraborty's population amalgamation test for the Dinaric capercaillie was consistent with the proposed glacial refugia/post-glacial migration scenario for the species [2], [23]. The Dinaric population could, therefore, be viewed as being composed of a few sporadically distributed descendants of *southern lineage* individuals populating the region during the last glacial maximum (LGM) and a majority of *boreal lineage* individuals outcompeting the *southern lineage* population during the post-glacial geographic and demographic expansion of the *boreal lineage*. Under this assumption, we tested only the *boreal lineage* portion (144 samples, i.e., 96.00%) of the Dinaric population (Dinarides-A). The mismatch distribution for Dinarides-A was unimodal and smooth (Figure 3C), Harpending's index and SSD were insignificant, Tajima's D and Fu's Fs were negative, the growth force parameter was high and statistically significant (Table 6), which is consistent with its proposed demographic expansion.

The Rhodopes-Rila group had a multimodal and rough mismatch distribution (Figure 3D) consistent with old stationary populations. The long-term stationary status of the Rhodopes-Rila group was further supported by a high and statistically significant Harpending's raggedness index and an insignificant growth force parameter value.

Generally, statistically significant negative values of both Tajima's D and Fu's Fs test are considered a sign of population expansion. It should be noted that at least in the case of Tajima's D test, statistically significant negative values for Dinarides and Dinarides-A were within test confidence interval (−1.769–2.089 for a sample of 150 individuals at 95% confidence limit), so agreement with neutrality could not be rejected based solely on Tajima's D test [30], [31].

## Discussion

All sampled individuals from the Slovenian Alpine populations belong to the *boreal lineage*, with the highest haplotype diversity (15 different haplotypes) of the sampled populations. Eight (53%) of the 15 Alpine haplotypes were novel, previously not described. It should be noted that haplotype TuB39 (also found in the Dinarides) was not considered to be novel since it is assumed to be identical to haplotypes Tu7 [2] and TUMF [21]; the authors of the former publication did not publish this haplotype sequence data, while the authors of the latter used different PCR primers, making only part of our sequence useful for comparison. The smooth and unimodal mtDNA CR mismatch distribution and numerous low frequency haplotypes are consistent with an expanding population. The significant and high positive growth force parameter and significant negative Fu's Fs value also suggest population expansion. Actual Western Capercaillie number counts for the Slovenian Alps indicate that the population has been in decline since the 1960s [14]. The discrepancy between demographic parameters estimated from the analysed genetic marker and actual trends observed in the field can be explained by the fact that results inferred from mtDNA CR data are more indicative of the long term population history, with more recent population dynamics not yet significantly affecting the mtDNA CR make-up of the studied population, or that population changes have not been drastic enough to be detectable on the level of mtDNA CR. Similar demographic discrepancy has been reported previously for Alpine/Central European and Finnish Western Capercaillie, which exhibit population expansion inferred from mtDNA data but have actually suffered a decline in recent decades [21], [23]. The expanded population status for the *boreal lineage* according to the mtDNA data has been discussed as a result of its rapid post-glacial expansion to its current habitat range [2], [21], [23] and is supported by the star-like topology of the MSN trees [32].

The Dinaric populations are composed of representatives of both lineages, with the *boreal lineage* being distinctly dominant, with 96.00% of the samples. Tu11 is the single most dominant haplotype in the Dinarides, representing 68.67% of the samples. Such a prevalence of a single haplotype is in stark contrast to the neighbouring Alpine and Rhodopes-Rila populations, where the most dominant haplotypes represent only 24.51% (Tu11) and 36.07% (T2), respectively. The above-mentioned single-haplotype-prevalence and overall low genetic diversity/high homogeneity could be the result of severe population decline and isolation. Similarly low genetic diversities have been reported for isolated capercaillie populations in the Black Forest and Thuringia, where a severe reduction in numbers has occurred in recent decades [22].

The Bulgarian Rhodope and Rila mountains population is composed of both lineages, with *southern lineage* individuals (6 different haplotypes) representing 90.16% of the sample. The multimodal and rough mtDNA CRI mismatch distribution and the presence of few frequent haplotypes suggest that the Rhodopian population is in demographic equilibrium (stationary) or declining. Similar demographic trends have been reported for Cantabrian and Pyrenean populations, which are also composed of both lineages [23]. A recently published paper on numbers and distribution of Bulgarian capercaillie showed that the population is currently stationary [19], which is in agreement with our analyses. High pairwise F_ST_ values, the results of Monmonier's maximum difference algorithm and AMOVA support claims that the Bulgarian capercaillie is very likely a different subspecies (*T. urogallus rudolfi*) from the Alpine and Dinaric capercaillie (*T. urogallus major*). The results of these population differentiation tests are consistent with the proposed subspecies distribution [20].

The presence of *southern lineage* individuals in the Dinarides confirms our hypothesis that at least the SE Dinarides served as a glacial refugium for capercaillie during the last glacial maximum (LGM). *Southern lineage* individuals were discovered in the central and south-eastern parts of the mountain range (i.e., Bosnia and Herzegovina and Montenegro) and appear to be randomly distributed, since we were unable to identify any local predominantly *southern lineage* demes. We hypothesize that *southern lineage* individuals could be considered to be descendants of the capercaillie population existing in the area during LGM and out-competed by the *boreal lineage* sometime during the period following the LGM and expansion of the latter lineage. On this assumption, the Dinarides can be identified as the north-westernmost contact zone of the two lineages in the Balkans. The historic demographic expansion of the *boreal lineage* in the region is supported by the results of demographic analyses in this study. Our findings extend the known species LGM habitat range in the Balkans [2] north-westward to the central Dinaric region (BiH). Despite being recognised as one of major glacial refugia [33], the Balkans, and the Dinarides in particular, have remained poorly studied in the field of avian phylogeography. To date, the Dinarides have been reported in the literature as a contact zone between genetic lineages of only a small number of vertebrate species, such as Martino's Vole (*Dinaromys bogdanovi*) [34], the Bank Vole (*Clethrionomys glareolus*) [35], the Common Newt (*Triturus vulgaris*) [36] and *saxatilis* and *graeca* subspecies of Rock Ptarmigan (*Alectoris graeca*) [37]. A pattern of long-term demographic stability of southern latitude (refugial) populations and demographic expansion of northern latitude populations observed in Western Capercaillie [2], [21], [23,this study] has also been reported for birds from other continents, such as MacGillivray's Warbler (*Oporornis tolmiei*) [38] in North America, and interpreted in relation to Pleistocene glacial cycles.

It is plausible that the *southern lineage* was more abundant in the Dinarides in the more recent past and that the current low abundance is the result of a severe population decline (population bottleneck or near-bottleneck conditions) with subsequent population recovery through limited numbers of surviving Dinaric individuals and *boreal lineage* migrants from the Alps. An alternative explanation of the presence of *southern lineage* individuals in the Dinarides is the possibility of a historic exchange of migrants from the Rhodope-Rila-Pirin massif through the Osogovo-Belasica mountain range in the east, and the West Vardar-Pelagonia mountain range and Šar Mountains in the central and western part of FYR Macedonia.

The presence of capercaillie in Macedonia has not been factually confirmed since at least 1946 [39, Velevski M. pers comm 2010]; however, some suitable capercaillie habitat patches reportedly still existed in Macedonia between 1946 and 1956 [39]. High levels of genetic differentiation between Dinaric and Bulgarian populations and the presence of unique Dinaric *southern lineage* haplotypes suggest that these two populations have been effectively isolated for a considerable time. It is plausible that the constant anthropogenic pressure, which perhaps started already in pre-antiquity, caused the destruction of suitable forest habitats in most of Macedonia and consequently the interruption of a Dinarides-Macedonia-Bulgaria capercaillie habitat corridor.

In addition to current population decline, historic records of land use suggest a possibility that severe population reduction conditions also existed for capercaillie in Slovenia between the 15^th^ and 18^th^ centuries [40]. Unregulated clearing of virgin forests for pasture and the use of wood for iron ore processing led to a severe decrease in forest cover of potentially suitable Western Capercaillie habitat areas in Slovenia [14], [41]. Available data suggest that by the late 18^th^ century, forests were fragmented and reduced to 20% in the Alps and 30% in the Dinarides, with a significant reduction of Western Capercaillie populations [14]. A significant reduction of forest cover and Western Capercaillie populations by the 18^th^ to early 19^th^ centuries have also been reported for other alpine countries [8].

Changes towards more sustainable land use practices introduced in the 19th century led to a gradual increase in forest cover and capercaillie population numbers in Slovenia, which reached a peak between 1910 and 1930 [12], [14]. The present forest cover for the Slovenian Alps and Dinarides is between 70% and 80% [14]. Genetic analyses of mtDNA CRI for Slovenian alpine populations did not reveal any evidence of a reduction of genetic diversity due to any current or historic reduction of population numbers. Nucleotide and haplotype diversities were comparable to those reported for the Alpine metapopulation by other authors [2], [22], [23]. We hypothesize that, due to the inaccessibility of higher altitude alpine forests, in which capercaillie populations are most numerous and stable [10] enough habitat remained undisturbed and sufficient inter-deme connectivity was maintained, enabling viable capercaillie populations to survive and recover without an excessive decrease in genetic diversity. Another factor that may have contributed to the successful recovery of Slovenian Alpine populations in the past was the possibility of attracting migrants from neighbouring Austrian and Italian populations.

Assuming a similar reduction of forest cover in the 15^th^ to 18^th^ centuries in the Dinaric region in a southeast direction from Slovenia, we propose a scenario explaining the present low level of genetic diversity, derived from mtDNA CRI data analyses for the Dinaric population. Mature forest habitats in the Dinarides are characterised by a higher proportion of deciduous species and much lower abundance of the essential bilberry [42] than those in the Alps [14]. Distribution of suitable Western Capercaillie habitats in the Dinarides is naturally patchier and more fragmented than in the Alps with consequently lower overall population densities [14], [17], [43]. Suitable vegetation (abundance of conifers and acidophilic ericaceous shrubs) – mostly limited to mixed silver fir-beech stands with Norway spruce appearing only at specific topological features, such as sinkholes, valleys and depressions, with a cooler microclimate due to temperature inversion – is generally more abundant towards the lower end (800–1200 m a.s.l.) of the capercaillie altitude range (800–2000 m a.s.l. [12], [18], [44], [45]). As a consequence, Western Capercaillie populations in the Dinarides are generally found at lower altitudes thus being closer to human settlements and potentially more affected by land use, hunting and also exposed to higher predator densities [14], [16] than populations in the Alps where suitable vegetation, dominated by Norway spruce, is more abundant at higher altitudes (1200–1600 m a.s.l.) [14]. Due to habitat specificities (i.e., lower Index of Habitat Suitability than the Alps) [14], the effect of anthropogenic habitat destruction and disturbance was probably much more pronounced in Dinaric Western Capercaillie populations, possibly leading to more severe population reduction and fragmentation, resulting in the low observed genetic diversity in populations at the present time.

The above-mentioned differences between the Alpine and Dinaric habitats are not reflected significantly in mtDNA CRI sequence diversification of respective populations, as evident from low pairwise F_ST_ values and Monmonier's maximum difference algorithm results. Historic records [14], the absence of unique Dinaric haplotypes in the Slovenian and Croatian Dinarides, the number of shared haplotypes and their frequencies all suggest that a high level of connectivity existed between Alpine and Dinaric populations in the past. The mountains in western Slovenia served as a contact zone through which the exchange of migrants between the two populations was possible. According to Adamič [18] and Čas [10], [14], the functional connectedness of Alpine and Dinaric populations was lost after 1960, so the Dinaric capercaillie population can effectively be considered to be isolated. South-eastern Dinaric populations appear to be more differentiated from the Alpine, as evidenced by the presence of unique Dinaric haplotypes. It is, of course, not excluded that analysis of more variable genetic markers, such as microsatellites, would reveal additional genetic diversification of Alpine and Dinaric populations.

Our phylogenetic analyses regrouped the haplotypes discovered in the studied populations into two distinct haplogroups (clades). The existence of two distinct capercaillie lineages in Eurasia has been demonstrated previously by microsatellite [11], mtDNA [2], [23] and combined mtDNA and Cytochrome B analyses [22]. Different scenarios have been proposed concerning the evolution of the two lineages: (i) Duriez et al. [2] hypothesized that both lineages already existed in pre-glacial Eurasia, with the *southern lineage* expanding throughout Europe, at least to Romania and Bulgaria, while the *boreal lineage* expanded in Asia and North-Eastern Europe. During the LGM, the lineages retreated to separate refugia: Iberia and the Balkans for the *southern lineage*, South Asia or Beringia for the *boreal lineage*. After the LGM, the *boreal lineage* expanded and replaced the *southern lineage* in most of Eurasia (perhaps being more competitive) with the latter surviving only in Cantabria, the Pyrenees and, to a lesser extent, in the Balkans – all at the extreme southern edge of the species habitat range. (ii) Rodríguez-Muñoz et al. [23] proposed an alternative scenario, based on the paraphyletic topology of the maximum likelihood tree, suggesting an ancestral status of the *southern lineage*. They discussed the possibility of the *southern lineage* being broadly distributed in pre-glacial Europe, with distribution becoming fragmented with the advance of glaciation. The *southern lineage* may have survived in Mediterranean refugia, with new *boreal lineage* haplotypes evolving in the Balkans or Italian refugia and expanding throughout Eurasia following climate warming and the retreat of the ice sheet. The latter authors also predicted the existence of southern lineage haplotypes in the Balkans or Italy, a hypothesis confirmed by Duriez et al. [2] and our study. Both scenarios explain the existence of the observed contact zone between the lineages in the Pyrenees.

**Figure 4:**
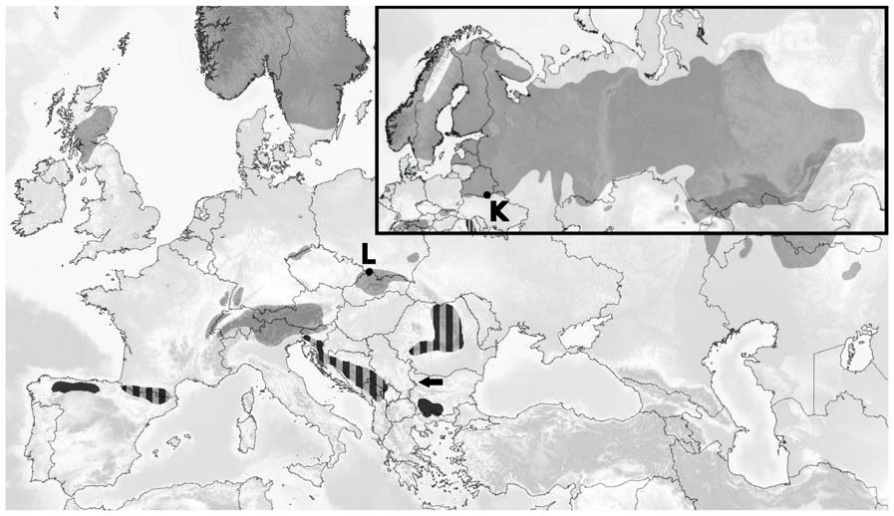
Western Capercaillie (*Tetrao urogallus*) distribution map. The areas shaded light gray mark the *boreal lineage* range, the dark gray areas mark predominantly *southern lineage* populations, striped areas are mixed/contact-zone populations. The black arrow marks the isolated west Balkan mountain range population [1], [2], [16], [19]. K – sampling location in Pripyat, Belarus; L – sampling location in the Carpathian Mountains, Poland.

Both MSN and ML trees in our study also suggest the ancestral position of the *southern lineage*. Of particular interest is that, according to ML and the MSN tree, the two lineages are linked via haplotypes found in Bulgaria (i.e., T3 for the *boreal lineage*, discovered in our study, and Tu44 for the *southern lineage*, reported by Duriez et al. [2]). The existence of the two closest interlineage haplotypes (discovered to date) in the same locality (i.e., the Rhodope-Rila-Pirin mountain chain) perhaps offers more support to the capercaillie lineages evolutionary scenario proposed by Rodríguez-Muñoz et al. [23]. The colonisation of northern and western Europe by mtDNA lineages originating from the Balkans, forming contact zones with lineages from the Apennine and Iberian refugia, was reported for the European Grasshopper (*Chorthippus parallelus*) [46] and the Tawny Owl (*Strix aluco*) [47]. The assumption that the *boreal lineage* evolved in colder boreal-like habitats of glacial Eurasia, and as such was better adapted to and more competitive in similar climatic conditions, offers a possible explanation of why the *boreal lineage* was more successful than the *southern lineage* in colonizing Eurasia during successive habitat changes following the retreat of glaciation (see Figure 4 for a lineage distribution map). It should be noted that, due to low bootstrap support of the ML tree, the ancestral position of the *southern lineage* cannot be confirmed nor rejected. Manual inspection of jackknifed MSN trees, however, revealed (data not shown) that the *southern lineage* appeared ancestral to the boreal lineage in 76% and that haplotypes originating in the Balkans (not exclusively T3-Tu44) represented 74% of the closest or one of equally probable closest interlineage haplotype pairs.

Additional samples from the Italian Alps, the Balkans and other parts of SE Europe, and the southern edge habitat range in Asia (Russia and Kazakhstan) should be analysed to gain more accurate and reliable results in relation to the geographic and evolutionary origin of lineages of Western Capercaillie. Furthermore, evidence is mounting that, in addition to traditionally recognised Mediterranean glacial refugia, smaller glacial refugia also existed further north in the Carpathians and the Carpathian Basin in Central and SE Europe [35], [36], [48]–[50], opening new avenues of thought when considering the origin of both Western Capercaillie lineages.

Accurate calculation of the capercaillie lineage divergence time would provide a criterion for estimating the validity of the proposed scenario that the *boreal lineage* evolved from the *southern lineage* in the Balkans during the period of major glaciations. Rodríguez-Muñoz et al. [23] dated the capercaillie lineages divergence time at 171,000 years before the present, based on the grouse mtDNA CR mutation rate estimated by Drovetski [51]. Lineage divergence time estimated by Rodríguez-Muñoz et al. [23] generally fits the timeline of the Riss-Saalian glacial period in Europe. It should be noted that caution should be asserted when interpreting the grouse mutation rate and divergence time estimate, as the molecular clock hypothesis for grouse mtDNA CR was rejected in the very same study conducted by Drovetski [51], as well as separately for *T. urogallus* mtDNA CR [2]. Furthermore, the results of a direct approach analysis of mtDNA mutation rates of humans [52], nematodes [53] and Adélie penguins [54] suggest significantly higher (actual) mutation rates than those derived from an indirect evolutionary-phylogenetic approach. Although direct extrapolation of these findings to capercaillie mtDNA CR is not possible, it nevertheless raises questions about the validity of mtDNA CR based dating of evolutionary events. For the aforementioned reasons, we give no estimates of capercaillie lineage divergence time.

Analysis of a mitochondrial DNA control region, although a reliable genetic marker for inferring the long term demographic history, phylogeography and basic phylogeny of the studied species, also has its limitations. In particular, analyses of mtDNA CRI have limited power to detect more recent demographic and evolutionary events, such as recent bottlenecks, interlineage hybridisations and additional, sub-lineage, diversifications. Analyses of additional genetic markers, such as nuclear microsatellites, would be required to improve the significance and reliability of the estimates presented in this study, foremost on the level of population genetics and higher resolution phylogenetic analyses.

Similar to most western, central and southern European capercaillie populations [1], all of the populations sampled in this study also suffered a decline in the past. The Bulgarian population was reduced by 22% from 1965 to 1984, with the extinction of the species in some areas [19], [55]. Since the 1990s, however, the negative trend has been reversed and Bulgarian capercaillie populations are reportedly stable at present [19]. In the Slovenian Alps, capercaillie numbers dropped by 35% between 1980 and 2000 and are still declining [14]. Dinaric capercaillie have probably suffered the most severe decline in the region, with a reported 58% reduction in the number of individuals in the Slovenian Dinarides from 1980 to 2000 [14] and a 50% decline in Bosnia and Herzegovina from 1992 to 2004 [43]. Although official information is lacking for some of the studied Dinaric countries, the decline appears to be continuing [14], [16], [17], [43]. The capercaillie is listed as endangered in national Red Lists in all of the sampling countries, though regulated seasonal hunting is still allowed in Bosnia and Herzegovina, Kosovo and Bulgaria [16], [19], [43], [56]–[59].

Principal threats to capercaillie in the region include: (i) human disturbance of habitat areas due to mountain tourism and traditional picking of wild berries, mushrooms and herbs, (ii) destruction of old forest habitats by forestry and farming operations, (iii) climate change and forest development to more deciduous and thus less suitable stands, (iv) high predator densities and (v) collisions with wire fences for the Slovenian Alps and Dinarides [12], [15]. The principal threats in the rest of the Dinarides (Croatia, Bosnia and Herzegovina, Serbia, Kosovo, Montenegro) are similar but also include poaching and wildfires [16], [17], [43], [45], [60]. The poaching of males during the mating season in spring is considered an important additive cause of mortality [61] in the central and south-eastern Dinarides [16], [17], [43]. The main reasons for the decline of capercaillie numbers in Bulgaria in the past were similar to threats listed for the Slovenian Alps and Dinarides, but did not include collisions with wire fences [19].

The first large-scale sampling of the Bulgarian population, to our knowledge, conducted in this study, revealed that it is composed predominantly of *southern lineage* individuals, similar to Cantabrian and Pyrenean populations [2], [22], [23] and, as such, is unique and distinct in the region. Our findings also extend the known range of the *southern lineage* in the Balkans to the Dinarides. As evident from the distribution map (Figure 4), *southern lineage* populations and mixed populations cover only a minute fraction of the entire species habitat range and are located at its southern edge. Duriez et al. [2] proposed that these populations, i.e. Cantabrian, Pyrenean and Balkans (Bulgarian and Romanian) should be considered to be a single Evolutionary Significant Unit (ESU) forming a *southern lineage* that diverged from the remaining Eurasian populations (*boreal lineage*) and that each should be considered to be a separate conservation Management Unit (MU). We propose that the three populations sampled in our study be considered to be distinct MUs, due to differences in habitat characteristics and different genetic lineage composition. An effort, however, should be made to coordinate conservation strategies for the Slovenian Alpine and the entire Dinaric population, since they once formed a connected metapopulation. Although Bulgarian populations are reportedly stable [19], long-term conservation plans should be devised and implemented to ensure capercaillie survival at this *southern lineage* core area in the Balkans.

Due to the low genetic diversity revealed in this study and its isolated status, the current Dinaric population appears to be particularly vulnerable to further habitat fragmentation, loss of intra-population connectivity and population size reduction. Continuing population decline may lead to a further reduction in genetic diversity, with potentially severe consequences in terms of the fitness of the population and, ultimately, its survival. Loss of functional connectedness with the Alpine population is probably affecting both but will probably have a more negative long-term effect on the Dinaric population. Currently, the Dinaric population is estimated to be no more than 1400–1500 individuals [1], [14], [16], [43]. With continuing decline and inability to draw migrants from neighbouring populations, the Dinaric metapopulation is at risk of breaking into functionally disconnected populations, which would be smaller than the proposed *minimal viable capercaillie population size* of 500 [62] to 1000 individuals [11].

General conservation measures in the region should focus on preservation of suitable habitat areas, sustainable forestry and agricultural practices and landscape management, effective enforcement of a hunting ban and poaching and predator control. A particular effort should be made to secure and possibly increase connectivity between local populations through adapted forest management ensuring revitalisation and expansion of habitat corridors (towards a more stable metapopulation structure). The re-establishment of the connectedness of the Alpine and Dinaric populations in Slovenia would be of particular importance for the long-term survival of the capercaillie in the Western Balkans region.

If a potential reintroduction of individuals to areas of extinction in the Balkans were to be considered, special care should be taken in the origin of reintroduced individuals (i.e., *southern* vs. *boreal* lineage). Until the remaining Balkan populations are analysed (Albania, Greece, Macedonia, Balkan Mountains in Bulgaria and Serbia), we propose that Balkan populations should be treated as mixed populations composed of individuals from both lineages. Furthermore, no *Tetrao urogallus* reintroduction efforts in the Balkans should include only the *boreal lineage* but should also include the *southern lineage*, which appears to be more flexible and better adapted to forest stands with a higher proportion of deciduous trees [63]–[65]. Lineage-balanced reintroductions might be of particular importance in the light of global climate change and associated change in forest stands towards a higher proportion of broadleaf species [12], [60], [66]. The proportion of each lineage in reintroduced populations should, however, be consistent with the composition of natural populations in the region. In line with genetic rescue theory, the introduction of unrelated individuals from carefully selected populations should result in the reduction of the high frequency detrimental alleles characteristic of endangered populations exhibiting high genetic loads or inbreeding depression, and should consequently increase their fitness and viability [67]. On the other hand, the possibility should be considered that Western capercaillie populations in the Balkans may have evolved specific adaptive traits maintained under selective pressure of the local environment that cannot, necessarily, be detected by analyses of neutral genetic markers, especially mtDNA CRI. Rapid short-time-span microevolution of certain traits in birds has been reported previously [68], [69]. Thus it is possible that, despite an initial general increase in genetic diversity, even lineage-balanced reintroductions of individuals from different ecoregions (such as Scandinavia for the *boreal lineage* and the Cantabrian Mountains or Pyrenees for the *southern lineage*) could in fact decrease the fitness of populations under rescue by introduction of suboptimal traits. Further issues associated with reintroduction efforts include the risk of introduced individuals and their offspring outcompeting the endangered native population. It is thus vital to closely monitor the effects of introduced individuals on the population under rescue. Genetic rescue is probably the only viable solution to assure the survival of the endangered Western Capercaillie populations in the Balkans, such as the small and isolated population in the western Balkan Mountains (Figure 4).

## Materials and Methods

We collected a total of 319 samples (Table 2) of Western Capercaillie (*Tetrao urogallus*) from sites covering the species habitat in the Slovenian Alps, Dinarides (Slovenia, Croatia, Bosnia and Herzegovina, Montenegro, Serbia), and Rhodope and Rila Mountains (Bulgaria) (Figure 2), including a few from Poland and Belarus (Figure 4). Most samples were collected non-invasively on the ground (faeces and feathers) with the exception of 54 gizzard and liver tissue samples taken from birds shot legally by sport hunters in Bosnia and Herzegovina, 1 feather sample from Serbia taken from a legally shot trophy and 61 feather and tissue samples from Bulgaria taken from birds shot legally by sport hunters. To minimise the possibility of repeated non-invasive sampling of the same individuals the following precautionary steps were taken: (i) each lek site was sampled only once; (ii) primarily only two non-invasive samples were collected at each lek site; (iii) faeces and feather samples were collected under roosting trees that were at least 100 m apart; (iv) when possible, additional discriminating factors were considered, such as the diameter of droppings, feather colour and pattern, etc. No Western Capercaillie was harmed or killed directly due to research presented in this paper.

Faeces samples were lyophilized and stored in sealed plastic bags with silicagel at −20°C. Tissue samples were preserved in absolute ethanol in sealed vials and stored at −20°C until analysis. For phylogenetic analysis, we also used 51 mitochondrial DNA control region I (mtDNA CRI) sequences retrieved from Genbank (46 sequences of *T. urogallus* and 5 sequences of Black-billed Capercaillie (*Tetrao parvirostris*) [2], [21], [23], [70]), as indicated in Table 2.

Total DNA from faeces was extracted with the PowerSoil DNA isolation kit (MoBio Laboratories, Inc.), DNA from feathers and tissue samples was extracted with the UltraClean Tissue and Cells DNA kit (MoBio Laboratories, Inc.). Faeces samples (100 mg dry weight) were first cut into smaller pieces using sterile scissors and rehydrated with 2× volumes of DNA/RNA-free sterile dH_2_O prior to DNA extraction. Tissue samples were shredded using sterile tweezers, residual ethanol and liquids were removed by vacuuming in the Vacufuge™ Concentrator 5301 (Eppendorf) and 25 mg (dry weight) of material used for extraction. Both tip and blood clot in the superior umbilicus of the feather shaft were used for DNA extraction [71]. DNA from all sample types was extracted following the manufacturer's protocols. For each batch of samples a negative control extraction containing no biological material was also performed to test for possible contamination during DNA extraction.

We targeted the first 435 nucleotides of the mitochondrial control region I (CRI), the most variable domain of the mitochondrial DNA (mtDNA) control region in grouses [72]. CRI was PCR amplified with forward primer GalF and reverse primer GalRi [2]. Fifty µl reactions were prepared containing 15 mM Tris-HCl pH 8.0, 50 mM KCl, 2.5 mM MgCl_2_, 50 µM of each dNTP, 0.6 µM of each primer, 1.5 U of AmpliTaq® Gold (Applied Biosystems) and 1–5 µl of DNA extract. Reactions were performed using Applied Biosystems GeneAmp® PCR System 9700 thermocyclers with a polymerase activation step (95°C, 10 min), 5 touch-down cycles (denaturation 94°C, 45 sec; annealing 60°C – 56°C, 45 sec; extension 72°C, 45 sec), 45 amplification cycles (denaturation 94°C, 45 sec; annealing 55°C, 45 sec; extension 72°C, 45 sec) and a final extension step (72°C, 10 min). To assess possible contaminations or other problems, each batch of PCR reactions also included a DNA extraction negative control, a PCR negative control containing DNA/RNA-free water instead of DNA extract and a positive control containing DNA extract from the same tissue sample from Bosnia and Herzegovina. PCR products were resolved in 1.5% agarose gels and visualized by ethidium bromide staining. PCR yielding non-target amplification products were separated as above, target products were excised from the agarose gel and purified with Wizard® SV Gel and PCR Clean-up System (Promega). Sequencing was performed commercially at Macrogen Inc. (Seoul, Rep. of Korea) in both directions using amplification primers. Sequencing chromatograms were manually checked and corrected using FinchTV v1.4.0 software (Geospiza Inc.).

Sequences were aligned with ClustalX v2.0.12 [73]. We truncated the primer sequences and used only the first 413 bp, in order to have a homogenous dataset with sequences retrieved from Genbank (the excluded 22 bp did not include any variable sites). Phylogenetic analyses were conducted with PhyML v3.0 [74] using the Maximum Likelihood (ML) approach. The best-fit model of nucleotide substitution for ML analyses was selected under Corrected Akaike Information Criterion (AICc) with jModelTest v0.1.1 [74], [75] using ML optimized search strategy. ML analysis was conducted using the Subtree Prune and Regraft (SPR) search algorithm with 50 random starting trees. The robustness of the ML phylogenetic tree was assessed by bootstrapping [76], using 1000 replicates. A minimum spanning network (MSN) was constructed with Network v4.5.1.6 [77] according to the Median-Joining (MJ) algorithm with Maximum Parsimony (MP) post-analysis [78] and taking into account the Ti/Tv ratio as calculated by the jModelTest v0.1.1. To assess the support of MSN, a 100-replicate jackknife resampling with 50% deletion [76] was performed on the original dataset with Seqboot in the PHYLIP v3.69 package [79] and MSN trees reconstructed as described above. The jackknifed MSN trees were manually inspected and individual link and topology supports logged.

For population-level analysis, we pooled the populations into three geographical groups: Alpine, Dinaric and Rhodopes-Rila (Table 3). We used Arlequin v3.1.1 [80] for estimating population nucleotide diversities – π [81], [82], haplotype diversities – H [81], observed homozygosity – *Hom*_OBS_ [29], estimates of population parameter theta – Θ_S_ [83], Θ_Π_ [82], Tajima's D [31] and Fu's Fs [30]. Mismatch distributions were calculated for assessment of the demographic status of populations and their fit to expected values under demographic expansion [84], [85], [86], [87] evaluated according to associated sums of squared deviations – SSD and Harpending's raggedness index – r_H_ [88]. The present time population parameter theta – Θ_Pres_ [89] (Θ_Pres_ = 2·N_eF_·μ, N_eF_ is the effective female population size, μ is the mutation rate per site per generation) and population growth force parameters – g [90] were calculated for each population using Lamarc v2.1.5 [91]. The likelihood search strategy used included 3 replicates, each with 15 initial chains with 5% burn-in sampling 2000 genealogies at a sampling increment of 50 and 2 final chains sampling 25000 genealogies at a sampling increment of 50. All searches were run with simultaneous heating at two different temperatures. The statistical significance of the growth force was assessed according to the rule of inclusion/exclusion of zero in 95% confidence intervals taking into account the Bonferroni correction.

Population pairwise genetic distances (F_ST_ [92], [93]), exact test of population differentiation and pairwise inter- and intra-population distances were also calculated, according to the best-fit-model of nucleotide substitutions for our dataset, calculated by jModelTest v0.1.1.

In order to detect possible genetic barriers and their locations between or within the studied populations, we applied Monmonier's maximum difference algorithm [94] as implemented in Barrier v2.2 [95]. Each sampling location (UTM coordinates) was assigned a polygonal neighbourhood according to Voronoi tesselation and triangulated according to Delauney triangulation. The genetic distance matrix between samples at the studied locations, as used by Barrier v2.2, was calculated in Arlequin v3.11 using the Tamura-Nei model with a T_i_/T_v_ ratio according to the jModelTest v0.1.1.

We used Arlequin v3.11 for computing analysis of molecular variance (AMOVA), assuming Tamura-Nei distances with a T_i_/T_v_ ratio according to jModelTest v0.1.1. For AMOVA, the sampled populations were assigned to two groups according to Barrier v2.2: the first including all sampled Alpine and Dinaric demes and the second including Rhodopes-Rila demes.
